# Supplementary material for: Expression of β-globin by cancer cells promotes cell survival during blood-borne dissemination
Source: Nat Commun. 2017 Feb 9;8:14344. doi: 10.1038/ncomms14344 (PMC5321792; doi:10.1038/ncomms14344)
Supplement: Supplementary Information — Supplementary Figures and Supplementary Tables [file ncomms14344-s1.pdf]

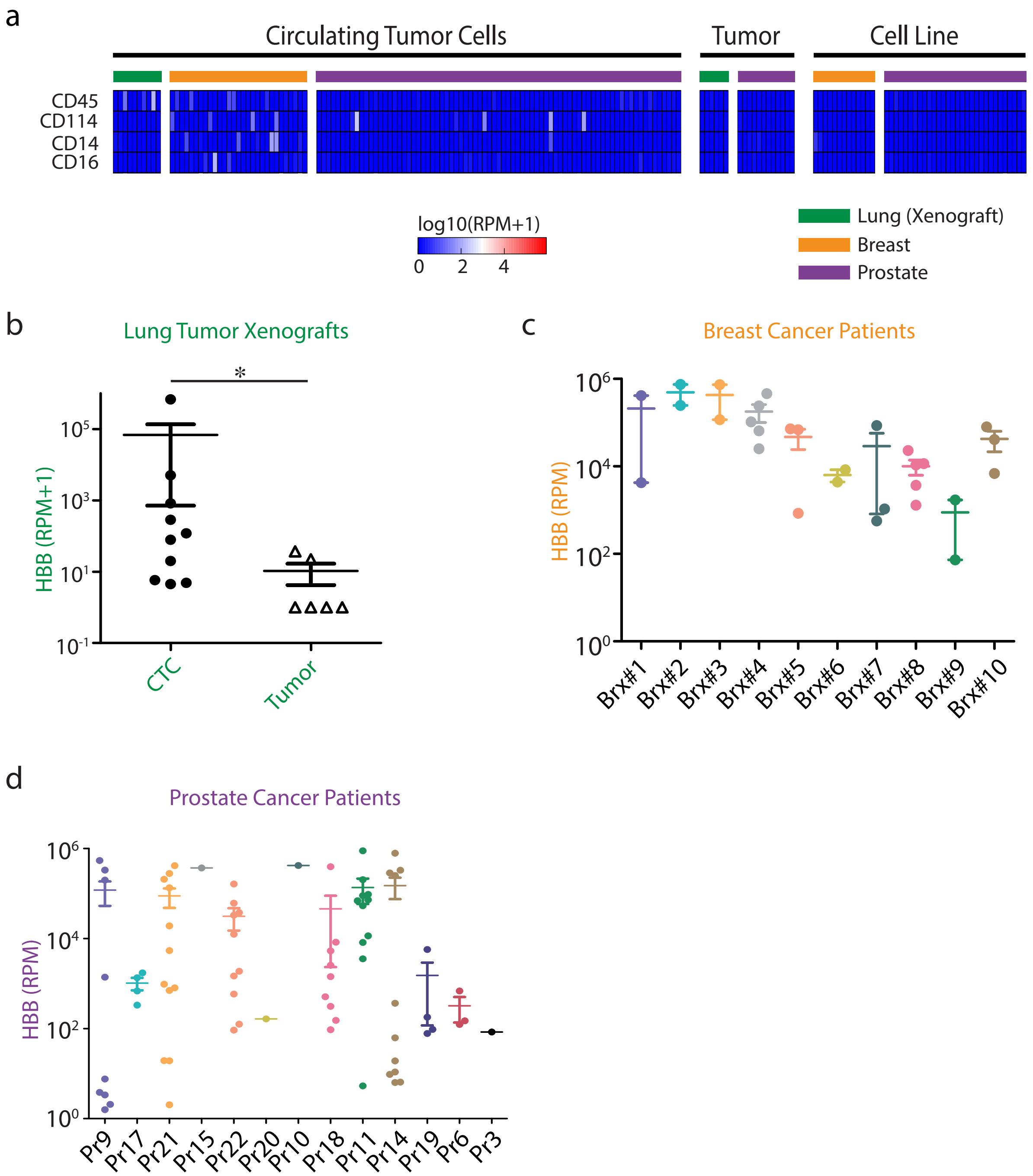

Supplementary Figure 1. *HBB* expression in circulating tumor cells in multiple types of human cancer
(a) Heatmap showing that the hematopoietic lineage markers (*CD45, CD114, CD14, CD16*) are absent or minimally expressed in lineage-confirmed CTCs from different cancer types. (b) Scatter plot showing increased expression of *HBB* in single CTCs or CTC-clusters isolated from the blood of lung tumor xenograft models, compared with single primary tumor cells. (c) Scatter plot showing the expression of *HBB* in single CTCs or CTC clusters across 10 breast cancer patients. (d) Scatter plot showing the expression of *HBB* in single CTCs across 13 prostate cancer patients. Data are represented as mean ± SEM. * denotes $p<0.05$ (t-Test).

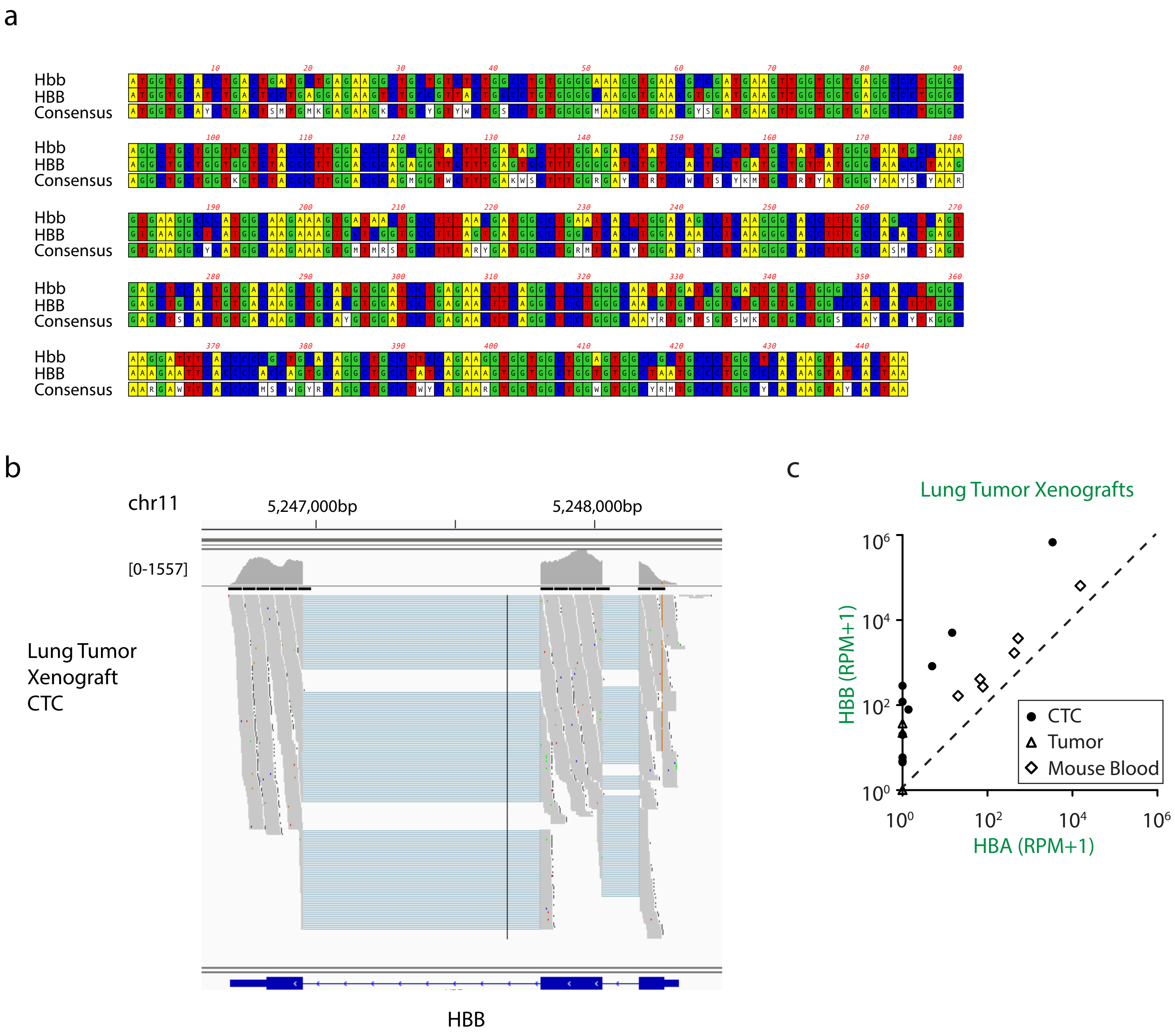

Supplementary Figure 2. Tumor specific origin of *HBB* in circulating tumor cells from xenografts
(a) Comparison of human *HBB* and murine *Hbb* gene sequences showing multiple nucleotides that can be used to distinguish human *HBB* and murine *Hbb* reads in NextGen Seq. (b) A snapshot of an IGV window showing the *HBB* reads of a single CTC from a lung xenograft model aligned to the *HBB* gene locus in human genome. (c) Scatter plot demonstrating the specific upregulation of *HBB* but not *HBA* in CTCs from lung xenografts. Mouse blood samples acquired from the lung xenograft model, aligned to mouse genome mm10, serves as a control, demonstrating comparable expression of *HBB* and *HBA* in blood cells.

a

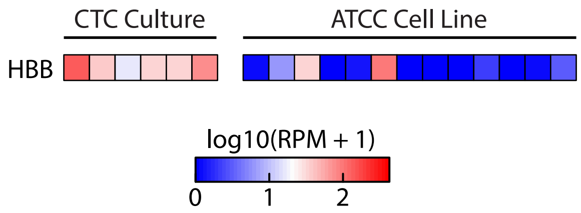

b

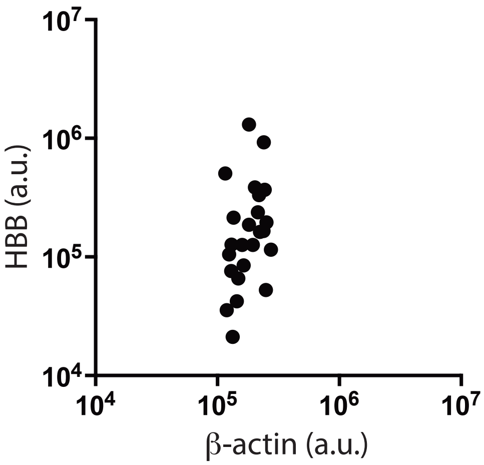

Supplementary Figure 3. Expression of *HBB* in cultured breast CTCs
(a) Heatmap showing the expression of *HBB* in cultured breast CTC lines and standard ATCC breast cell lines.
(b) Scatter plot showing a dynamic range of *HBB* protein levels in multiple cultured breast CTC lines (established from 6 breast cancer patients, at multiple time points during the treatment) detected by quantitative proteomics. A housekeeping gene β-actin was used as control showing the tight expression window among different samples.

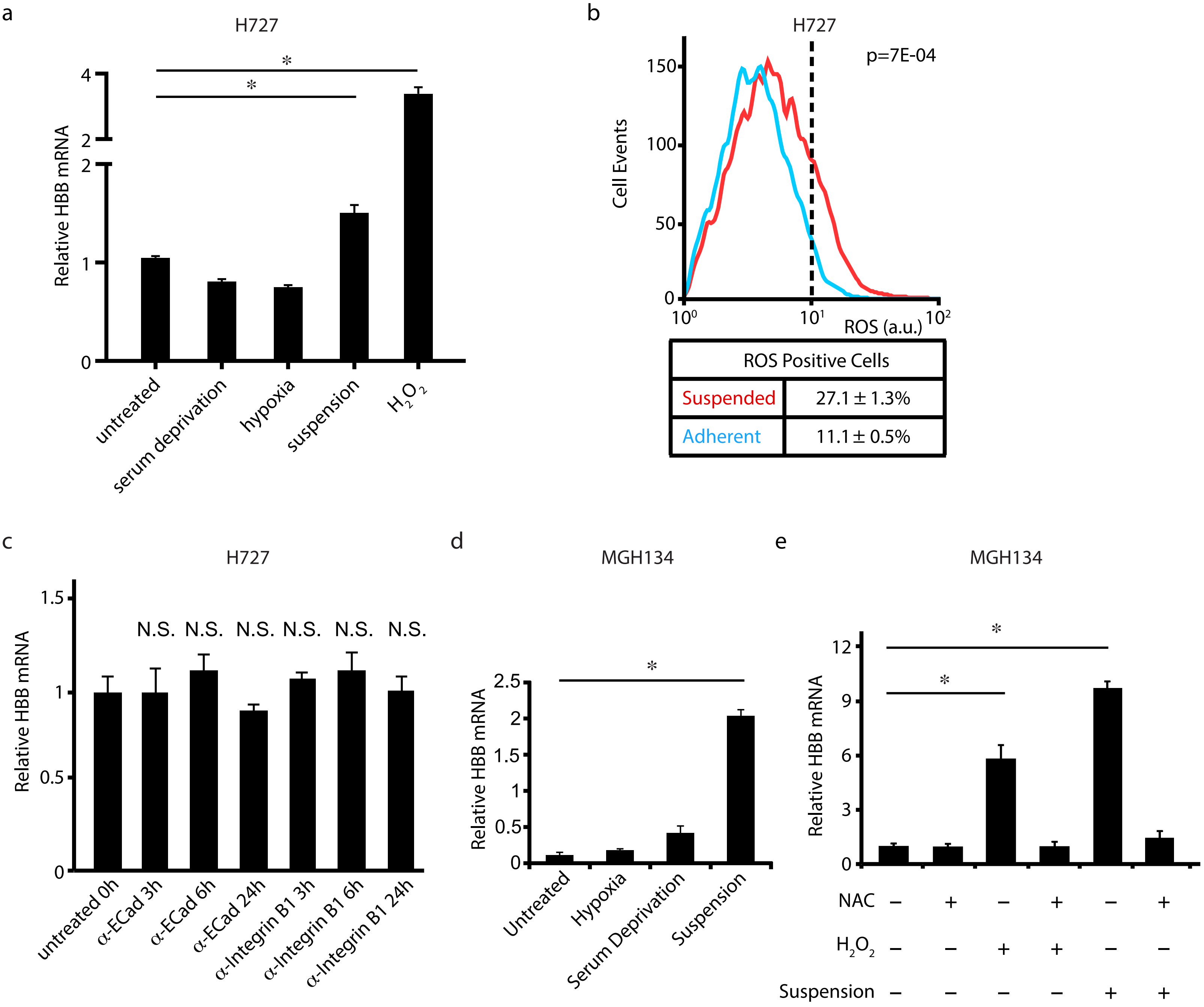

Supplementary Figure 4. Induction of *HBB* in H727 and MGH134 cells
(a) Bar graph showing *HBB* expression in H727 cells under various stress-related environmental stimuli. (b) Flow cytometric analysis showing increased percentage of ROS positive H727 cells after 24-hour culture in suspension, as detected by H2DCFDA dye. (c) Bar graph showing normalized *HBB* mRNA levels in H727 cells after the treatment with integrin or E-cadherin blocking antibody in a 24-hour time course experiment. (d) Bar graph showing normalized *HBB* mRNA levels in MGH134 cells under multiple stress conditions. (e) Bar graph showing normalized *HBB* mRNA levels in MGH134 cells under suspension and following H_2O_2 treatment. Pretreating cells with 1mM NAC blocks the induction of *HBB* by suspension or H_2O_2 treatment. All data are represented as mean ± SD. n= 3; * denotes a statistical significance at P<0.05 (t-Test).

a

GeneSymbol	chandran2007a	taylor2010_new	varambally2005
ATF5	0.540804491	0.188460582	0.653521174
ASCL1	0.170741346	0.184628596	0.726453251
MTA1	0.415950856	0.178953928	0.7357646
ONECUT2	0.369520336	0.277543656	0.735569676
MBD3	0.313013327	0.23324292	0.701883629
HOXB6	0.34251757	0.273489169	0.589624433
MYBL2	0.333360059	0.484158302	0.803181676
SOX4	0.284981807	0.146606414	0.752839712
FOXM1	0.440039695	0.361838289	0.802208221
ZNF273	0.337571627	0.224205857	0.884414109
MLXIP	0.405814111	0.21592261	0.619839501
EZH2	0.363024648	0.122011651	0.765122705
FOXA2	0.300022172	0.177660539	0.875635881

correlation r

0 1.0

b

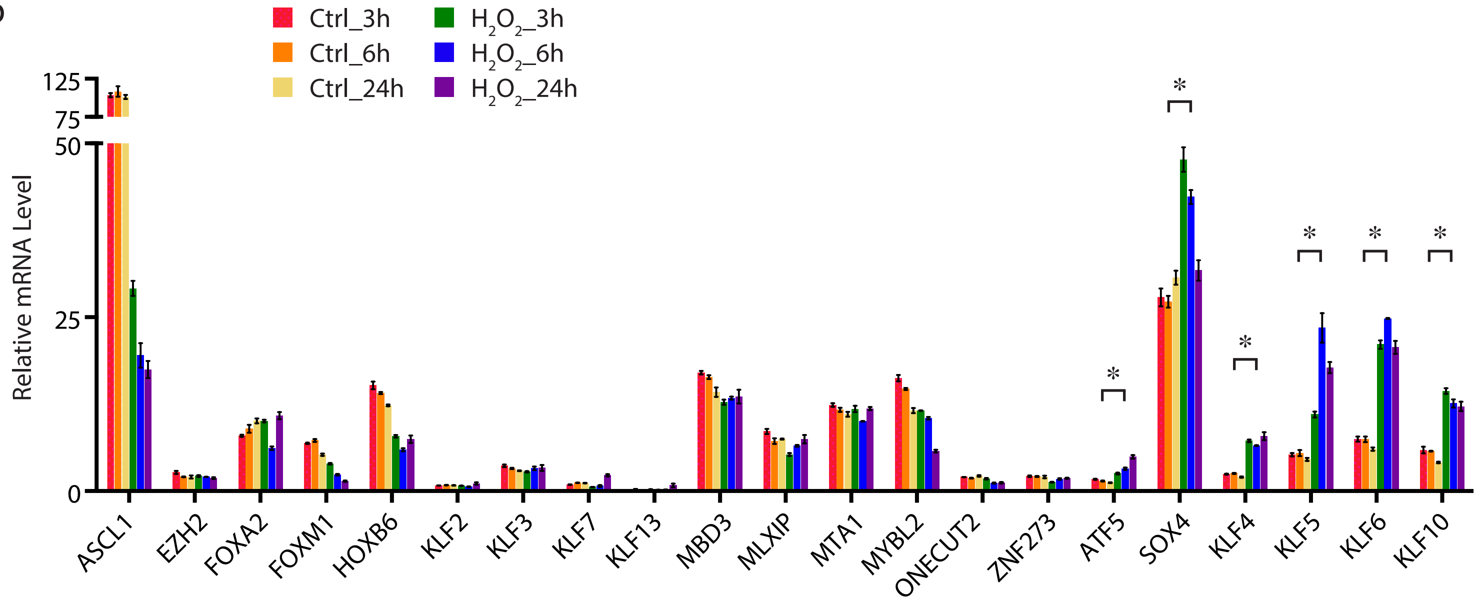

c

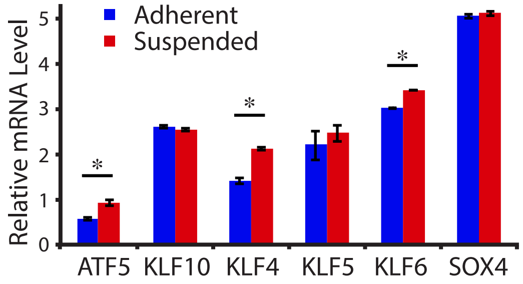

Supplementary Figure 5. Identification of transcriptional regulators that mediate *HBB* induction

(a) Heatmap of the correlation coefficients between each candidate transcription regulator and *HBB* in three publicly available datasets. (b) Bar graph showing normalized mRNA levels of all candidate transcription regulators in untreated and hydrogen peroxide-treated H727 cells at various time points. (c) Bar graph showing normalized mRNA levels of *ATF5, KLF10, KLF4, KLF5, KLF6* and *SOX4* in H727 cells under adherent or suspension conditions. All data are represented as mean ± SD. n= 3; * denotes a statistical significance at P<0.05 (t-Test).

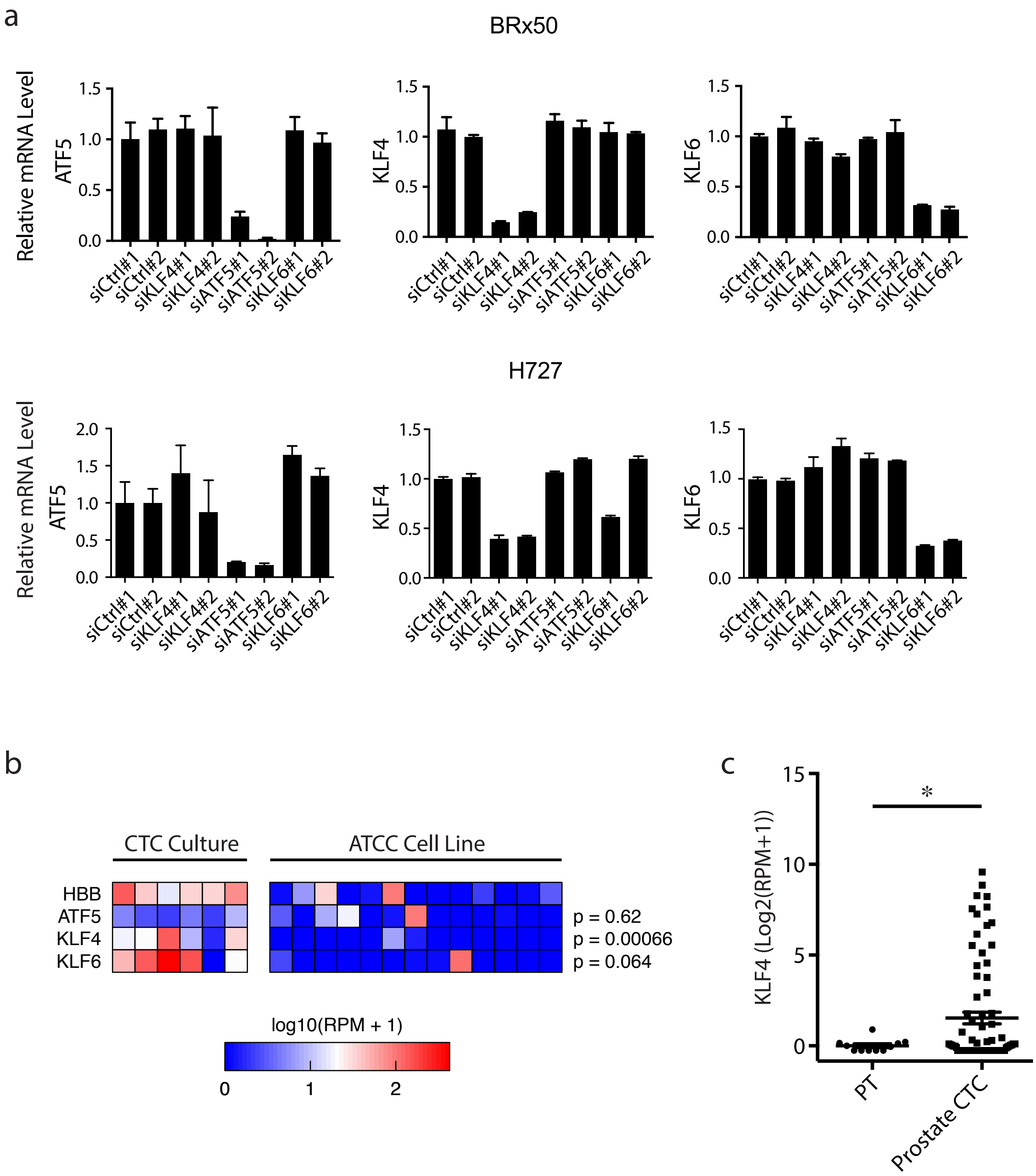

Supplementary Figure 6. Identification of *KLF4* as a transcriptional factor that mediates *HBB* induction
(a) Bar graph showing the knockdown efficiency of siRNAs against *ATF5, KLF4* or *KLF6* in BRx50 and H727 cells. Data are represented as mean ± SD. n= 3. (b) Heatmap showing the expression of *HBB, ATF5, KLF4* and *KLF6* in cultured breast CTC lines and ATCC breast cancer cell lines. p values for the correlation coefficients between each gene and *HBB* are indicated on the right. (c) Scatter plot showing the expression of *KLF4* in prostate CTCs compared with primary prostate tumor samples. Data are represented as mean ± SEM. * denotes a statistical significance at P<0.05 (t-Test).

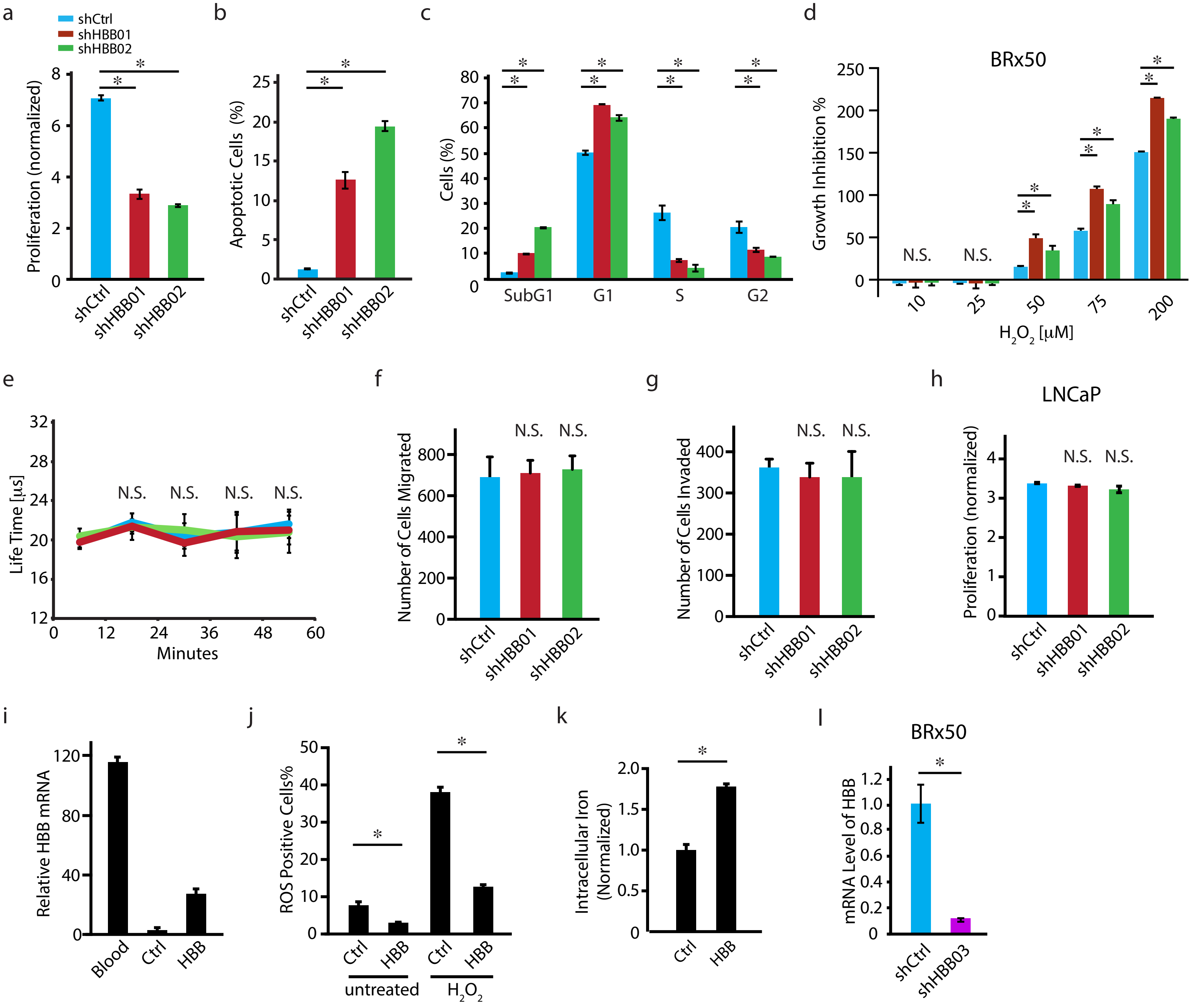

Supplementary Figure 7. Pro-survival function of *HBB* against oxidative stress in cancer cells

(a) Bar graph showing that *HBB* depletion reduces the proliferation of H727 cells (measured at day 5). (b) Bar graph showing increased apoptosis in *HBB*-depleted H727 cells (Annexin V apoptosis assay) at the same timepoint. (c) Bar graph shows that *HBB* depletion increases the percentage of H727 cells in subG1 and G1 phases, accompanied by decreases in S and G2 phases. (d) Bar graph showing increased sensitivity to various doses of H_2O_2 in HBB-depleted BRx50 cells compared with control cells. (e) Line graph showing the lifetime (μs) of an intracellular O2 sensor probe over a period of 60 minutes. No difference in intracellular O2 level was observed between control and *HBB*-depleted H727 cells. (f-g) Bar graph showing unchanged migration and invasive potential in *HBB*-depleted H727 cells in Boyden chamber assays. 20ng/ml HGF was used as chemoattractant. (h) Bar graph showing that proliferation of LNCaP cells is not affected by *HBB* shRNAs. (i) Real-time PCR showing the relative *HBB* mRNA levels in H727 at baseline (Ctrl) or following stable expression of ectopic *HBB*. Expression levels of *HBB* in a blood sample are shown for comparison. (j) Bar graph showing that ectopic overexpression of *HBB* in H727 cells reduces intracellular ROS under basal conditions and following treatment with hydrogen peroxide. (k) Bar graph showing increased total iron within H727 cells overexpressing *HBB* compared with control cells. (l) Bar graph showing the knockdown efficiency of *HBB* shRNA#3 in a cultured breast CTC line BRx50. All data (a-l) are represented as mean ± SD. n=3; * denotes a statistical significance at $P<0.05$.

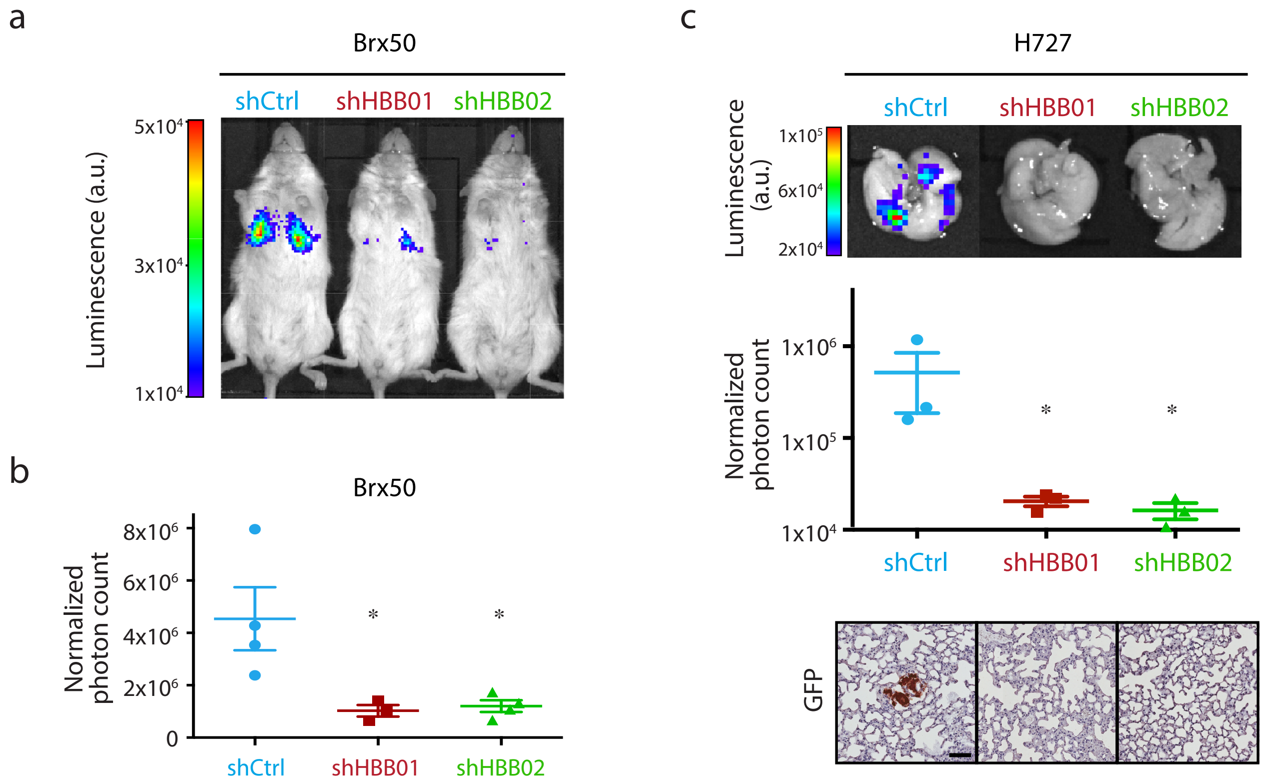

Supplementary Figure 8. Pro-survival function of *HBB* against oxidative stress in BRx50 and H727 cells in vivo
(a) Representative images of mice intravenously injected with BRx50 cells expressing control shRNAs or shRNAs against *HBB* at the end time point day 33 (luminescence by IVIS imaging). (b) Scatter plot showing quantitative normalized lung photon counts for each mouse at day 33. (c) Mouse intravenous injection model showing impaired metastatic potential of H727 cells upon *HBB* depletion. Top: representative images of isolated whole lung, at day 63 following intravenous injection of H727 cells expressing either control or *HBB*-targeting shRNAs (luminescence by IVIS imaging). Middle: Scatter plot showing normalized lung photon counts for each mouse at day 63. Bottom: representative images of the lung tissue immunohistochemistry using GFP antibodies. Scale bar, 100μm. Data are represented as mean ± SEM. n=3; * denotes a statistical significance at $P<0.05$ (t-Test).

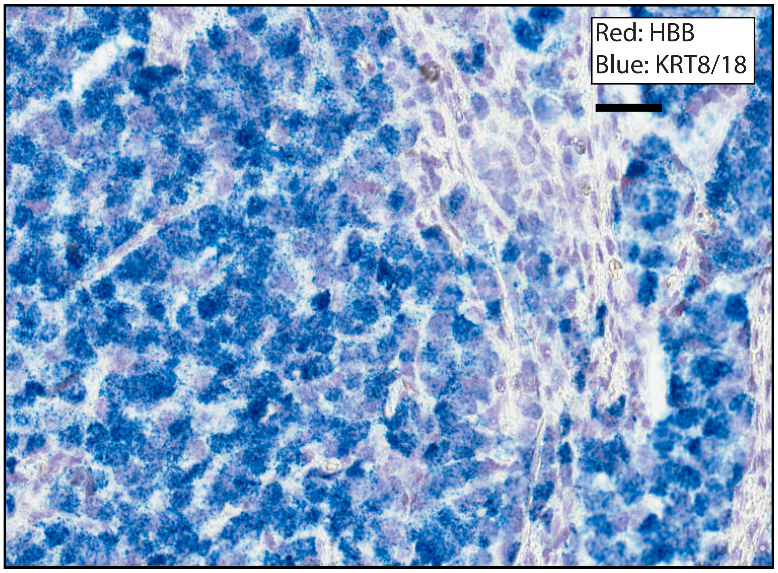

PCa Primary Tumor (N=9 pts)

Supplementary Figure 9. RNA in-situ hybridization assay of *HBB* in localized prostate tumors
Representative micrograph (40x) of RNA-in situ hybridization assay showing *HBB* and *KRT8/18* expression in localized prostate tumors Scale Bar, 25μm.

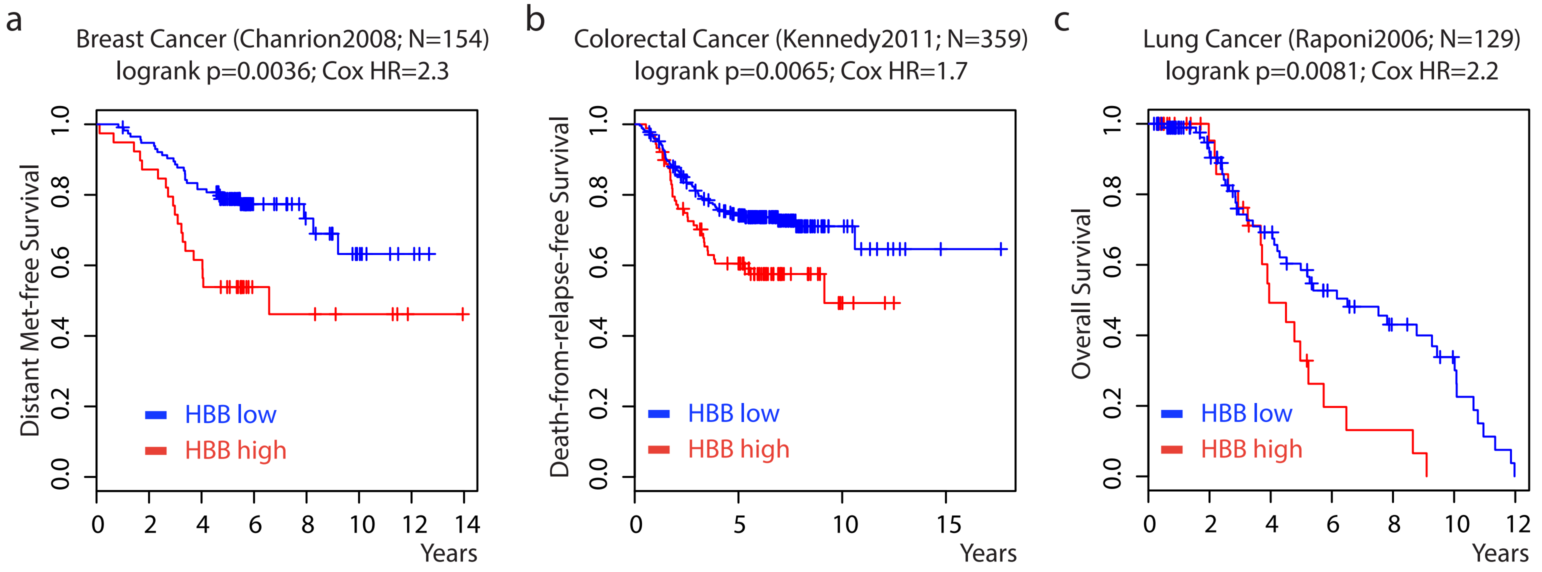

Supplementary Figure 10. Correlation of *HBB* expression with poor patient prognosis in multiple cancer types
(a) Kaplan-Meier survival curve of breast cancer patients showing expression of *HBB* is associated with occurrence of distant metastasis (n=154, p=0.0036). (b) Kaplan-Meier survival curve of colorectal cancer patients showing higher expression of *HBB* is associated with poor death-from-relapse-free survival (n=359, p=0.0065). (c) A Kaplan-Meier survival curve of lung cancer patients showing higher expression of *HBB* is associated with poor overall survival (n=129, p=0.0081). Red: patients with *HBB* expression above upper quartile. Blue: below upper quartile.

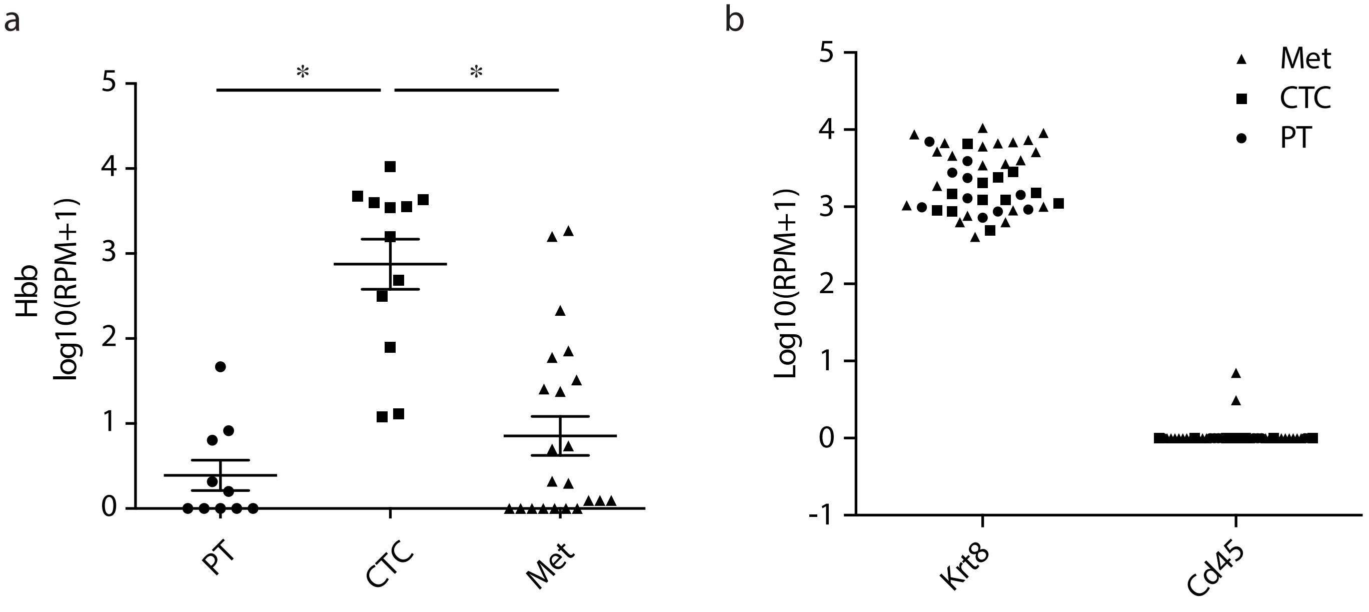

Supplementary Figure 11. RNA levels of *Hbb* in single CTCs and primary and metastatic prostate tumor cells
(a) Scatter plot showing *Hbb* mRNA expression in single CTCs and single primary and metastatic tumor cells from a metastatic prostate cancer mouse model. Data are represented as mean ± SEM. * denotes $p<0.05$ (t-Test). (b) Scatter plot showing abundant expression of epithelial lineage marker *Krt8* and minimal or absent expression of the hematopoietic lineage marker *Cd45* in single cells shown in A.

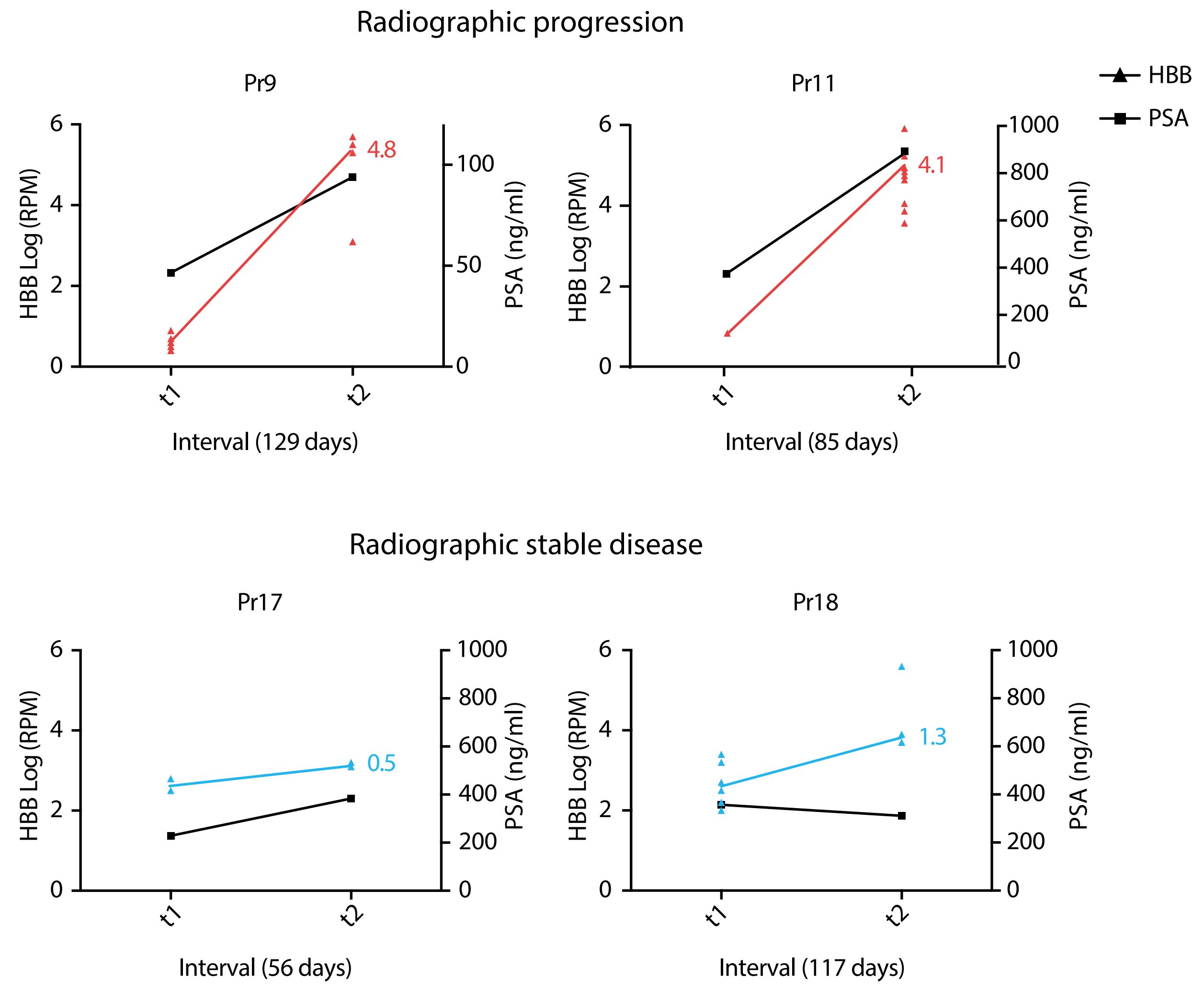

Supplementary Figure 12. Increased *HBB* expression in CTCs from CRPC patients with disease progression
Graphs showing changes in *HBB* levels in CTCs from serial blood draws from four different CRPC patients (Numbers on the right of the line indicate the log fold change of *HBB*). PSA levels are plotted according to the right Y axis. Intervals are indicated at the X axis.

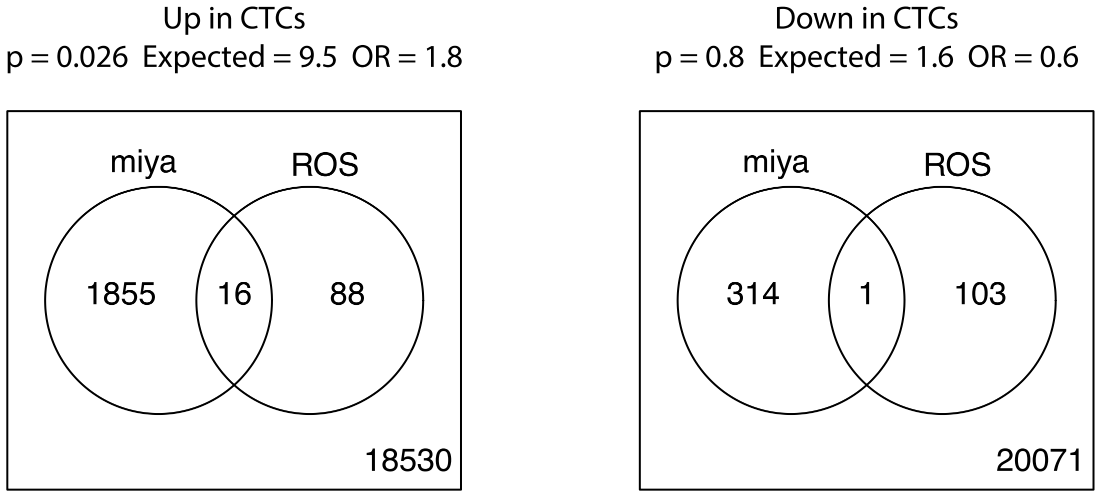

Supplementary Figure 13. Antioxidant genes differentially expressed in prostate CTCs and primary tumors
Venn diagram showing the intersection of genes that are upregulated or downregulated in prostate CTCs versus primary tumor and genes that are involved in ROS regulation.

Supplementary Table 1. List of datasets used for Kaplan-Meier survival analysis

Type	Data set name	Suvival type	p-value	Hazard ratio	FDR	Pubmed ID	Data source
breast	chanrion2008	Overall survival	0.004440365	2.373981843	0.09477495	18347175	GEO GSE9893
breast	pawitan2005	Overall survival	0.043025676	0.393252865	0.301179731	16280042	GEO GSE1456
breast	sabatier2010	Relapse-free survival	0.076414616	0.605049853	0.382073078	20490655	GEO GSE21653
breast	vantveer2002.new	Distant met-free survival	0.114456465	1.651899316	0.421256667	11823860	*
breast	chin2006	Overall survival	0.123694826	1.620340824	0.421256667	17157792	**
breast	schmidt2008	Distant met-free survival	0.141479895	0.551782129	0.421256667	18593943	GEO GSE11121
breast	zhou2007	Relapse-free survival	0.144430857	3.82E-09	0.421256667	17407600	GEO GSE7378
breast	buffa2011	Distant met-free survival	0.167724267	0.682382936	0.4306528	21737487	GEO GSE22219
breast	ivshina2006	Relapse-free survival	0.17226112	0.703111574	0.4306528	17079448	GEO GSE4922
breast	loi2008.new	Distant met-free survival	0.237729979	0.308557463	0.554703285	18498629	GEO GSE9195
breast	wang2005.new2	Distant met-free survival	0.31657109	0.7924576	0.651764008	15721472	GEO GSE2034
breast	tcga_brca	Overall survival	0.40290278	0.838709085	0.717091172	23000897	http://cancergenome.nih.gov/
breast	sotiriou2006.new	Distant met-free survival	0.414527227	0.73449198	0.717091172	16478745	GEO GSE2990
breast	finak2008.new	Relapse-free survival	0.447551155	0.557435028	0.717091172	18438415	GEO GSE9014
breast	vandevijver2002.new	Overall survival	0.450743022	1.208305562	0.717091172	12490681	***
breast	vandevijver2002.new2	Overall survival	0.450743022	1.208305562	0.717091172	12490681	****
breast	minn2007.new	Distant met-free survival	0.524002986	0.610689942	0.764171021	17420468	GEO GSE5327
breast	ma2004	Distant met-free survival	0.608890784	0.788835566	0.841942569	15193263	GEO GSE1378
breast	li2010	Distant met-free survival	0.682601517	0.767691824	0.841942569	20098429	GEO GSE19615
breast	desmedt2007	Overall survival	0.688521832	1.128873265	0.841942569	17545524	GEO GSE7390
breast	bos2009.new	Distant met-free survival	0.738997649	0.942640297	0.841942569	19421193	GEO GSE12276
breast	minn2005.new	Distant met-free survival	0.986714593	0.993991634	0.989038922	16049480	GEO GSE2603
breast	symmans2010	Relapse-free survival	0.989038922	1.003778535	0.989038922	20697068	GEO GSE17705
colorectal	kennedy2011	Death from disease-free survival	0.006502282	1.723747679	0.09477495	22067406	Array Express E-MTAB-863 & E-MTAB-864
colorectal	staub2009	Overall survival	0.021494061	3.496972697	0.188073031	19399471	GEO GSE12945
colorectal	jorissen2009	Relapse-free survival	0.118887644	0.75986701	0.421256667	19996206	GEO GSE14333
colorectal	smith2010	Overall survival	0.497489493	0.847201652	0.757049229	19914252	GEO GSE17536,GSE17537
lung	raponi2006	Overall survival	0.008123567	2.152995479	0.09477495	16885343	GEO GSE4573
lung	takeuchi2006	Overall survival	0.057287803	0.572537915	0.334178849	16549822	GEO GSE11969
lung	tomida2009	Overall survival	0.259985574	0.672126181	0.568718443	19414676	GEO GSE13213
lung	lee2008	Relapse-free survival	0.738140756	0.909246473	0.841942569	19010856	GEO GSE8894
lung	zhu2010	Overall survival	0.794166111	0.905246142	0.868619184	20823422	GEO GSE14814
prostate	taylor2010.new	Overall survival	0.72709903	0.745855254	0.841942569	20579941	GEO GSE21034
prostate	sboner2010.v2	Overall survival	0.745720561	0.945991625	0.841942569	20233430	GEO GSE16560
prostate	long2014	Relapse-free survival	0.850310163	0.950163709	0.901844112	24713434	GEO GSE54460

* http://www.rii.com/publications/2002/vantveer.html http://www.nature.com/nature/journal/v415/n6871/suppinfo/415530a.html

** https://caarraydb.nci.nih.gov/caarray/publicExperimentDetailAction.do?expId=1015897589973255

*** http://www.rii.com/publications/2002/nejm.htm http://microarray-pubs.stanford.edu/wound_NKI/Clinical_Data_Supplement.xls

**** http://www.rii.com/publications/2002/nejm.htm http://microarray-pubs.stanford.edu/wound_NKI/Clinical_Data_Supplement.xls

Supplementary Table 2. Antioxidant genes upregulated or downregulated in prostate CTCs

Symbol	Name	Fold-change	p-value
HBB	Hemoglobin, beta	19.5	1.89582E-06
ALDH18A1	Aldehyde dehydrogenase 18 family, member A1	4.5	1.65554E-08
ALDH9A1	Aldehyde dehydrogenase 9 family, member A1	7.8	3.29345E-10
ATOX1	ATX1 antioxidant protein 1 homolog (yeast)	6.0	2.33945E-09
CAT	Catalase	2.1	6.91411E-05
CSDE1	Cold shock domain containing E1, RNA-binding	12.4	0.000139708
MPV17	MpV17 mitochondrial inner membrane protein	2.9	0.005447409
PNKP	Polynucleotide kinase 3'-phosphatase	2.4	1.25665E-05
PRDX1	Peroxiredoxin 1	24.4	3.21909E-05
PRDX2	Peroxiredoxin 2	11.5	0.008957268
PRDX3	Peroxiredoxin 3	6.2	6.89518E-08
PRDX4	Peroxiredoxin 4	17.4	9.32281E-11
PRDX5	Peroxiredoxin 5	3.6	0.001381065
PRDX6	Peroxiredoxin 6	16.4	0.000272133
RNF7	Ring finger protein 7	7.0	1.89227E-07
SOD1	Superoxide dismutase 1, soluble	17.7	0.002003694
VIMP	VCP-interacting membrane protein	3.0	7.3866E-06
ALDH1A3	Aldehyde dehydrogenase 1 family, member A3	0.12	0.01942927

Supplementary Table 3. Patient CTC samples with corresponding GEO accession ID

Prostate CTC Samples

GEO accession	Sample ID	GEO accession	Sample ID
GSM1660118	Pr10.2.2	GSM1660157	Pr18.1.5
GSM1660119	Pr11.1.1	GSM1660158	Pr18.1.6
GSM1660120	Pr11.2.2	GSM1660159	Pr18.1.7
GSM1660121	Pr11.3.1	GSM1660160	Pr18.1.8
GSM1660122	Pr11.3.2	GSM1660161	Pr18.2.1
GSM1660123	Pr11.3.3	GSM1660162	Pr18.2.2
GSM1660124	Pr11.4.1	GSM1660163	Pr18.2.3
GSM1660125	Pr11.4.2	GSM1660164	Pr19.1.2
GSM1660126	Pr11.4.3	GSM1660165	Pr19.1.3
GSM1660127	Pr11.4.4	GSM1660166	Pr19.1.4
GSM1660128	Pr11.4.5	GSM1660167	Pr19.1.5
GSM1660129	Pr11.4.6	GSM1660168	Pr20.1.1
GSM1660130	Pr14.1.1	GSM1660169	Pr21.1.1
GSM1660131	Pr14.2.1	GSM1660170	Pr21.1.10
GSM1660132	Pr14.2.2	GSM1660171	Pr21.1.11
GSM1660133	Pr14.2.3	GSM1660172	Pr21.1.12
GSM1660134	Pr14.2.4	GSM1660173	Pr21.1.2
GSM1660135	Pr14.2.5	GSM1660174	Pr21.1.3
GSM1660136	Pr14.2.6	GSM1660175	Pr21.1.4
GSM1660137	Pr14.3.1	GSM1660176	Pr21.1.5
GSM1660138	Pr14.3.4	GSM1660177	Pr21.1.6
GSM1660139	Pr14.3.5	GSM1660178	Pr21.1.7
GSM1660140	Pr14.3.6	GSM1660179	Pr21.1.8
GSM1660141	Pr15.1.1	GSM1660180	Pr21.1.9
GSM1660142	Pr17.1.1	GSM1660181	Pr22.1.10
GSM1660143	Pr17.1.2	GSM1660182	Pr22.1.11
GSM1660144	Pr17.2.1	GSM1660183	Pr22.1.12
GSM1660145	Pr17.2.2	GSM1660184	Pr22.1.2
GSM1660146	Pr9.1.1	GSM1660185	Pr22.1.4
GSM1660147	Pr9.1.2	GSM1660186	Pr22.1.5
GSM1660148	Pr9.1.3	GSM1660187	Pr22.1.6
GSM1660149	Pr9.1.4	GSM1660188	Pr22.1.7
GSM1660150	Pr9.1.6	GSM1660189	Pr22.1.8
GSM1660151	Pr9.3.1	GSM1660190	Pr22.1.9
GSM1660152	Pr9.3.6	GSM1660191	Pr3.1.1
GSM1660153	Pr9.3.7	GSM1660192	Pr6.1.1
GSM1660154	Pr9.3.8	GSM1660193	Pr6.1.2
GSM1660155	Pr18.1.1	GSM1660194	Pr6.1.5
GSM1660156	Pr18.1.4		

Breast CTC Samples

GEO accession	Sample ID
GSM1253375	Patient#1_CL#1
GSM1253376	Patient#1_SC#1
GSM1253377	Patient#2_SC#1
GSM1253378	Patient#3_SC#1
GSM1253379	Patient#4_SC#2
GSM1253380	Patient#5_SC#1
GSM1253381	Patient#2_CL#1
GSM1253382	Patient#5_SC#2
GSM1253383	Patient#6_CL#1
GSM1253384	Patient#6_SC#1
GSM1253385	Patient#7_CL#1
GSM1253386	Patient#7_SC#2
GSM1253387	Patient#8_SC#1
GSM1253388	Patient#9_CL#1
GSM1253389	Patient#9_SC#1
GSM1253390	Patient#7_SC#1
GSM1253391	Patient#5_CL#1
GSM1253392	Patient#4_SC#1
GSM1253393	Patient#4_CL#3
GSM1253394	Patient#4_CL#1
GSM1253395	Patient#4_CL#2
GSM1253396	Patient#3_CL#1
GSM1253397	Patient#10_SC#1
GSM1253398	Patient#10_CL#1
GSM1253399	Patient#10_CL#2
GSM1253400	Patient#8_CL#1
GSM1253401	Patient#8_CL#2
GSM1253402	Patient#8_SC#2
GSM1253403	Patient#8_SC#3

Supplementary Table 4. Disease Status of Prostate Cancer Patients with multiple blood draws

Patient	Stage	Disease status at time of CTC collection	PSA at first CTC collection (ng/mL)	PSA at last CTC collection (ng/mL)	Prior Treatments
Pr9	met.	CRPC, on abiraterone	46.52	93.9	ADT
Pr11	met.	CRPC, on abiraterone	391	898.3	ADT, bic, metformin, keto, cabo, doc
Pr17	met.	CRPC, on cabazitaxel	229.1	384.9	ADT, doc, abi
Pr18	met.	CRPC, on enzalutamide	356.8	311.3	ADT, sipT, metformin, cabo, doc

Supplementary Table 5. List of cell lines used in this study

Name	Type
BRx-07	CTC_breast
BRx-33	CTC_breast
BRx-42	CTC_breast
BRx-50	CTC_breast
BRx-61	CTC_breast
BRx-68	CTC_breast
HMEC	ATCC_breast
MCF10A	ATCC_breast
BT474	ATCC_breast
BT20	ATCC_breast
MDAMB231	ATCC_breast
ZR751	ATCC_breast
HCC1954	ATCC_breast
EFM19	ATCC_breast
UACC833	ATCC_breast
T47D	ATCC_breast
MCF7	ATCC_breast
MDAMB453	ATCC_breast
CAL51	ATCC_breast
PC3	ATCC_prostate
LNCaP	ATCC_prostate
VCaP	ATCC_prostate
DU145	ATCC_prostate
H727	ATCC_lung
MGH134	NSCLC
HEK293T	ATCC_embryonic kidney

Supplementary Table 6. List of datasets used for meta-analysis

Experiment ID	Reference
chandran2007a	Chandran et al. 2007 (PMID: 17430594)
chung2004	Chung et al. 2004 (PMID: 15144956)
grasso2012a<br>grasso2012b	Grasso et al. 2012 (PMID: 22722839)
lapoint2004a<br>lapointe2004b	Lapointe et al. 2004 (PMID: 14711987)
lee2010	Lee et al. 2010 (PMID: 20421545)
matsuyama2009	Matsuyama et al. 2010 (PMID: 20162577)
murat2008	Murat et al. 2008 (PMID: 18565887)
perou_superset.new2<br>perou_superset.new3<br>perou_superset.new4	Perreard et al. 2006 (PMID: 16626501)
phillips2006	Phillips et al. 2006 (PMID: 16530701)
roessler2015	Roessler et al. 2015 (PMID: 25552933)
taylor2010.new	Taylor et al. 2010 (PMID: 20579941)
varambally2005	Varambally et al.2005 (PMID: 16286247)
vecchi2008	Vecchi et al. 2008 (PMID: 17952122)
wuttig2010	Wuttig et al. 2010 (PMID: 22213152)
yu2012	Yu et al. 2012 (PMID: 23251464)

Supplementary Table 7. List of primers used in this study

Primer Name	Sequences (5' -> 3')
HBB_F	ACCTTTGCCACACTGAGTG
HBB_R	GCAAGAAAGCGAGCTTAGTGATAC
HBB_ChIP_F	TCCAACTCCTAAGCCAGTGC
HBB_ChIP_R	TCTGCCCTGACTTTTATGC
ASCL1_F	CGCGGCCAACAAGAAGATG
ASCL1_R	CGACGAGTAGGATGAGACCG
ATF5_F	TGGCTCGTAGACTATGGGAAA
ATF5_R	ATCAACTCGCTCAGTCATCCA
EZH2_F	GTACACGGGGATAGAGAATGTGG
EZH2_R	GGTGGGCGGCTTTCTTTATCA
FOXA2_F	GGAGCAGCTACTATGCAGAGC
FOXA2_R	CGTGTTCATGCCGTTCATCC
FOXM1_F	ATACGTGGATTGAGGACCACT
FOXM1_R	TCCAATGTCAAGTAGCGGTTG
HOXB6_F	GTGCTCCACTCCGGTCTAC
HOXB6_R	GTAACGTGTGTATGTCTGGCG
KLF10_F	CAGACCTGTTACACCAGTATC
KLF10_R	GGGTTCAAAGTCAGAAGGAC
KLF13_F	CGGCCTCAGACAAAGGGTC
KLF13_R	TTCCCGTAAACTTTCTCGCAG
KLF2_F	TGCCGTCCTTCTCCACTTTC
KLF2_R	AAGTCCAGCACGCTGTTGAG
KLF3_F	TACACAAGTCACCTCCAGC
KLF3_R	TGTCCTCTGTGGTTCGATCC
KLF4_F	CCTGGACCTGGACTTTATTC
KLF4_R	GGATCGGATAGGTGAAGCTG
KLF5_F	ACCTGGTCCAGACAAGATG
KLF5_R	TCTACGACTGAGGCACTGTC
KLF6_F	GAGCCCTGCTATGTTTCAG
KLF6_R	GTTCGGATTCCTCCTTTTTCTC
KLF7_F	TGGACATCTTGCTCTCTCG
KLF7_R	ATGAGGTCACTGCGTTGAG
MBD3_F	CTGAGCACCTTCGACTTCCG
MBD3_R	CCGGCTGCTTGAAGATGGA
MLXIP_F	TGGTGTTGAAGAATGCCCG
MLXIP_R	AGGATGCTGCTGTGGAACTC
MTA1_F	CATCAGAGGCCAACCTTTTCG
MTA1_R	GCACGTATCTGTCGGTGGTC
MYBL2_F	CTTGAGCGAGTCCAAAGACTG
MYBL2_R	AGTTGGTCAGAAGACTTCCCT
ONECUT2_F	GGAATCCAAAACCGTGGAGTAA
ONECUT2_R	CTCTTTGCGTTTGCACGCTG
SOX4_F	GACCTGCTCGACCTGAACC
SOX4_R	CCGGGCTCGAAGTTAAAATCC
ZNF273_F	ATGGTAGCCAAACCCCCAGTTG
ZNF273_R	ATCCGCACTTTTACAGCC
